# Dose response test of patient-derived cancer organoids to irradiation

**DOI:** 10.3389/fonc.2025.1677172

**Published:** 2025-10-27

**Authors:** Haina Yu, Canfeng Lin, Fan Chen, Lifeng Chen, Weizhen Liu, Hualong Liu, Jing Li, Ye Yao

**Affiliations:** ^1^ Department of Medical Oncology, Jinjiang Municipal Hospital (Shanghai Sixth People’s Hospital Fujian), Quanzhou, China; ^2^ Department of Vascular Surgery, Quanzhou First Hospital Affiliated to Fujian Medical University, Quanzhou, China; ^3^ Department of Medical Oncology, Sun Yat-sen University Cancer Center, State Key Laboratory of Oncology in South China, Guangdong Provincial Clinical Research Center for Cancer, Sun Yat-sen University, Guangzhou, China; ^4^ Department of Hematology, Union Hospital, Tongji Medical College, Huazhong University of Science and Technology, Wuhan, China; ^5^ Department of Gastrointestinal Surgery, Union Hospital, Tongji Medical College, Huazhong University of Science and Technology, Wuhan, China; ^6^ Department of Basic Medicine, Changsha Medical University, Changsha, China; ^7^ Department of Radiation Oncology, Wuzhou Red Cross Hospital, Wuzhou, China; ^8^ Department of Radiation Oncology, State Key Laboratory of Oncology in South China, Guangdong Provincial Clinical Research Center for Cancer, Sun Yat-sen University Cancer Center, Guangzhou, China

**Keywords:** organoids, precision medicine, rectal cancer, irradiation, dose response

## Abstract

**Background:**

Patient-derived cancer organoids emerged as an innovative model in basic and translational medicine research, as well as precision medicine. However, most radiation oncologists are still unaware of the value of organoids in radiation oncology research, especially in precision radiation oncology. The methods for assessing organoid cell death after irradiation are also undefined.

**Methods:**

Three organoid lines were successfully established from the surgical specimens of rectal cancer patients. Genetic characterization of rectal cancer organoids was conducted. The survival of rectal cancer organoids after X rays irradiation was evaluated by image-based analysis and cell counting kit-8.

**Results:**

The three organoid lines displayed diverse architecture and captured heterogeneity of the tumors. The most frequently mutated genes and pathways among rectal cancer tumors also presented in rectal cancer organoids. Image-based analysis validated organoid survival after X rays irradiation, while cell counting kit-8 failed. The survival curves from imaging analysis were more representative, with an initial linear slope, followed by a shoulder, and tending to become straight again. This was the first study to investigate the appropriate testing method of using organoids to study cancer radiosensitivity.

**Conclusion:**

Cancer organoids were easy to use for the research of radiobiology of cancer. Image-based analysis is more accurate than cell viability (tested by cell counting kit-8) for studying cancer radiosensitivity via organoids.

## Introduction

Although substantial progress in cancer treatments has been made, cancer remains a significant global public health problem and is the leading or second leading cause of death in many countries (1). Further improvement in cancer therapy rely on basic and translational cancer research. However, the efficacy of translating preclinical studies findings into clinical practice remains limited, mainly due to the poor performance of preclinical cancer models in recapitulating patient tumors (2). Moreover, conventional cancer therapies often work well in some patients but fail to show favorable results in others. Precision or personalized medicine has the potential to address this problem (3, 4). Unfortunately, precision medicine is hampered by several obstacles, particularly the lack of reliable personalized tumor models (5, 6).

Recently, patient-derived cancer organoids have emerged as an innovative model in basic and translational research, as well as in precision medicine (7–10). Since long-term culture of colonic and small intestinal epithelium became available (11), a variety of organoids derived from normal tissues and related cancer tumors have been established (12). Cancer organoids closely recapitulate the pathological, genetic, transcriptomic, and proteomic features of tumors (13, 14). Organoids have been used to investigate the roles of infectious factors in cancer development (15), mutational processes during tumorigenesis (16), genetic cancer modeling (17) and other aspects of cancer biology (14, 18). In translational and precision medicine, organoids have shown great potential for the establishment of living biobanks, drug screening and development, and clinical response prediction (7, 9, 10, 19).

The cancer models widely used in radiobiology include cancer cell lines, cancer cell spheres, patient-derived tumor xenografts (PDTX), and spontaneous and transgenic animal models (20). Recently, organoids derived from normal tissues have been used as a novel model to study radiation-induced normal tissue injury (21). Radiation oncologists have also begun to recognize the profound value of cancer organoids in cancer research, particularly in precision radiation oncology (20). Some radiation oncologists have even planned to use cancer organoids to measure personalized radiotherapy effects in co-clinical trials (22, 23). It is expected that the cancer organoids derived from patients will become widely used in basic and translational radiobiology research. However, organoid responses to irradiation have mainly been tested using cell viability assays (24–26), which were originally designed to determine cell death in cell lines. The analysis of organoid responses to irradiation should therefore be further improved and validated.

Here, we show that cancer organoids derived from patients with newly diagnosed rectal cancer represent an interesting and novel model for radiobiology and precision radiation oncology. The cancer organoid model is easy to use and allows personalized analysis. We also found that image-based analysis is more reliable for evaluating organoid responses to irradiation. Taken together, patient-derived cancer organoids might represent an ideal tool for basic and translational research in radiation oncology.

## Materials and methods

### Human specimens

Fresh surgical specimens of primary rectal carcinoma tumors were collected from patients treated at the Department of Gastrointestinal Surgery, Union Hospital, Tongji Medical College, Huazhong University of Science and Technology. Before radical resection of rectal cancer, no treatment was administered. Informed consent was obtained from all participants. All specimen collections and experiments were reviewed and approved by the Institutional Review Board of Union Hospital, Tongji Medical College, Huazhong University of Science and Technology.

### Surgical specimens processing

After harvesting, surgical specimens were immediately divided into three portions. One portion was immersed in cold PBS containing gentamicin/amphotericin B (Gibco, R01510) and normocin (InvivoGen, ant-nr-1), then transported on ice to the laboratory for tumor cell isolation and culture. The other two portions were promptly placed in 4% paraformaldehyde (Sigma-Aldrich, P6148) and liquid nitrogen for subsequent histopathological and molecular experiments, respectively.

### Isolation and culture of tumor cells

The rectal cancer tumor tissues were photographed and then washed in cold PBS containing streptomycin/penicillin (Gibco, 15140-122) for 5 min, repeated five times. Next, in a sterile dish on ice, the tissues were cut into small fragments and enzymatically digested in 8 mL digestion medium at 37 °C for 40 min on an orbital shaker. The digestion medium consisted of 20 μg/mL of 10 μM ROCK inhibitor Y-27632 (Sigma-Aldrich, Y0503), 500 U/mL collagenase IV (Sigma-Aldrich, C9407), 1% fetal bovine serum, and 7 mL DMEM medium (Gibco, C1199500BT). After shearing the digested tumor tissues using 5 mL and 10 mL pipettes, the suspension was centrifuged at 300–500 × g for 5 min. Finally, the tumor cells were collected and seeded into a 24-well cell culture plate (Costar, 3524) with Matrigel (Corning, 356231). After incubation in a culture incubator at 37 °C with 5% CO_2_ for 5–8 min, the cells were overlaid with 500 μL medium for culturing human rectal cancer organoids (RCOs).

### Rectal cancer organoids culture

Human RCOs were observed and photographed at appropriate time points and passaged every 1–2 weeks. Organoids were first gently pipetted out of Matrigel and mechanically sheared using a 1% bovine serum albumin (BSA)-coated pipette tip in cold PBS. The organoids were then centrifuged at 200–300 × g and washed several times until the Matrigel was completely removed. Finally, organoid fragments were seeded according to the previously described method, which was beneficial for new organoid formation. Organoids were cryopreserved under optimal conditions.

### Culture medium of rectal cancer organoids

The culture medium of RCOs was refreshed every 3 days. It consisted of Advanced DMEM/F12 medium (Gibco, 12634-010), 100 ng/mL Noggin (Sino Biological, 50688), 500 ng/mL R-spondin1 (Sino Biological, 11083-HNAS), 1% HEPES, 50 ng/mL EGF (Sino Biological, 50482-MNCH), 1% GlutaMAX, 1:500 gentamicin/amphotericin B (Gibco, R01510), 1:500 Normocin (InvivoGen, ant-nr-1), 500 nM A-83-01 (Tocris, 2939), 1:100 B27 supplement (Invitrogen, 17504-044), 1:50 N2 supplement (Invitrogen, 17502-048), 3 µM SB202190 (Sigma-Aldrich, S7067), 1 mM N-acetylcysteine (Sigma-Aldrich, A9165), 10 nM prostaglandin E_2_ (Sigma-Aldrich, P6532), 10 mM niacinamide (Sigma-Aldrich, N0636), and 10 nM gastrin (Sigma-Aldrich, G9145).

Cryopreservation of organoids was performed using serum-free cryopreservative medium (CELLBANKER™ 2, ZENOAQ, 170905). The resuscitation culture medium was supplemented with 10 μM ROCK inhibitor Y-27632.

### DNA extraction

Second-generation organoids after 3 days of culture were harvested under optimal conditions, and DNA was extracted using the SDS method according to the manufacturer’s instructions (Tiangen, DP705). The human peripheral blood lymphocyte isolation solution (TBD Science, HY2015) was used to isolate peripheral blood mononuclear cells (PBMCs). Similarly, germline DNA was extracted from frozen PBMCs using the SDS method. DNA degradation and contamination were assessed using 1% agarose gels.

### Whole exome sequencing and mutation analysis

DNA concentration was analyzed using a Qubit 2.0 Fluorometer (Invitrogen, USA). A total of 0.6 μg genomic DNA from each sample was used for library preparation. The Agilent SureSelect Human All Exon Kit (Agilent Technologies, CA, USA) was used to generate sequencing libraries according to the manufacturer’s instructions. The DNA libraries were sequenced to generate 150 bp paired-end reads using the Illumina HiSeq platform and then mapped to the human reference genome GRCh37 with maximal exact matches (15) using Burrows–Wheeler Alignment. MuTect2 (Genome Analysis Toolkit v3.8.0) with default parameters was used to identify somatic mutations by comparing the reference and organoid sequencing data. Annotation and effect prediction of mutations were performed using ANNOVAR. The genomic context of all somatic SNVs and the contribution of “mutational processes in human cancer” were analyzed in organoid samples using the MutationalPatterns R package, version 3.

### Organoids preparation for irradiation response

Rectal cancer organoids were collected and seeded into a 48-well cell culture plate (Costar, 3548) according to the method described previously. Each well was supplemented with 300 μL culture medium containing 15 μL Matrigel, and approximately 200 ± 50 organoids were seeded. Before irradiation testing, the fragments were allowed to develop into structurally complete and relatively large organoids, a process typically lasting 24–72 h.

### Cell viability assay

For the irradiation test, six irradiation doses (0, 2, 4, 8, 12, and 16 Gy) were selected to comprehensively characterize organoid radiosensitivity. This aligns with the classical dose–response curve approach commonly used in radiobiology for radiosensitivity assessment. This differs from our previously published 2020 Cell Stem Cell study (19), in which we generated dose–response curves for only a subset of the organoids and ultimately adopted an optimal single dose (8 Gy) with time–response curves. Those curves involved monitoring organoid survival over time post-irradiation, and that choice was primarily made to control research costs while still validating the method’s reliability for predicting clinical radiotherapy efficacy.

In the current study, we aimed to assess radiosensitivity using only three organoid lines—a small sample size that minimized cost concerns. Therefore, we returned to the multi-dose dose–response curve approach to ensure comprehensive and precise assessment. Specifically, 2 Gy and 4 Gy were selected as they represent the low-to-moderate dose range consistent with clinical fractionated radiotherapy regimens and help capture the “shoulder” region of the dose–response curve for assessing sublethal damage repair. Meanwhile, 12 Gy and 16 Gy cover the high-dose range to determine the organoids’ upper survival limit. Together, these doses captured the complete dose–response profile of the three organoid lines, comprehensively characterizing their radiosensitivity heterogeneity.

The organoids were exposed to X-rays (X-RAD 320, North Branford, CT, USA) at 246 cGy/min, 250.0 kV, 12.00 mA, and a source-to-sample distance (SSD) of 50 cm. The culture medium was replaced with fresh medium immediately after irradiation. Cell viability was assessed using the Cell Counting Kit-8 (CCK-8) assay on day 9 post-irradiation (0, 2, 4, 8, 12, and 16 Gy). On the ninth day after irradiation, the culture medium was aspirated and replaced with 300 μL DMEM medium (Gibco, C1199500BT) supplemented with a 1:11 dilution of CCK-8 solution (DOJINDO, CK04). The organoids were then incubated at 37 °C in a 5% CO_2_ atmosphere for 4 h. Finally, 100 μL of medium were transferred from each 48-well cell culture plate to a 96-well cell culture plate for detection. The optical density was measured using an ELx808 Absorbance Microplate Reader (BioTek, USA) and Gen5 Reader Control software, following the manufacturer’s instructions.

### Organoids cell death analysis based on image

Images of the organoids were captured every 3 days using a ZEISS microscope (ZEISS, Vert.A1) during the 15 days following irradiation. The number of viable organoids at day 15 post-irradiation (0, 2, 4, 8, 12, and 16 Gy) was quantified using ImageJ software, and the survival rate of the organoids was calculated. The organoid survival rate was used as an indicator of radiosensitivity, considering only the number of viable organoids. The criterion for determining organoid survival was the integrity of the organoid structure, which was independent of organoid size. Structural integrity (complete or disrupted structure) was evaluated by a trained observer, Dr. Ye Yao, who had extensive relevant experience. This principle for determining viable and nonviable organoids had been validated previously (19). We confirmed that morphologically intact organoids (defined by the above structural criterion) consistently exhibited positive staining with neutral red—a well-established marker of cellular viability—whereas structurally disrupted organoids showed no neutral red uptake. Organoid survival assessed on the basis of structural integrity accurately predicted the efficacy of chemoradiotherapy in rectal cancer. In conclusion, we believe that using organoid integrity for survival assessment is accurate; therefore, no additional staining was performed.

### Curve fitting of irradiation dose response data

Organoid dose–response analyses were conducted using GraphPad software. The irradiation dose–response curve fitting utilized the log[inhibitor] vs. normalized response–variable slope model with the equation Y = 100/(1 + 10^((LogIC50-X)*HillSlope)), and the single-hit multi-target model with the equation Y = 1-(1-exp(-k*x))^N (27, 28) for results from the CCK8 assay and image-based organoid survival, respectively.

### Microscopy and image analysis

Images of viable organoids were captured using a ZEISS microscope (ZEISS, Vert.A1) and analyzed with Image-Pro Plus 6.0 software.

## Results

### Establishment of rectal cancer organoids

**Figure 1:**
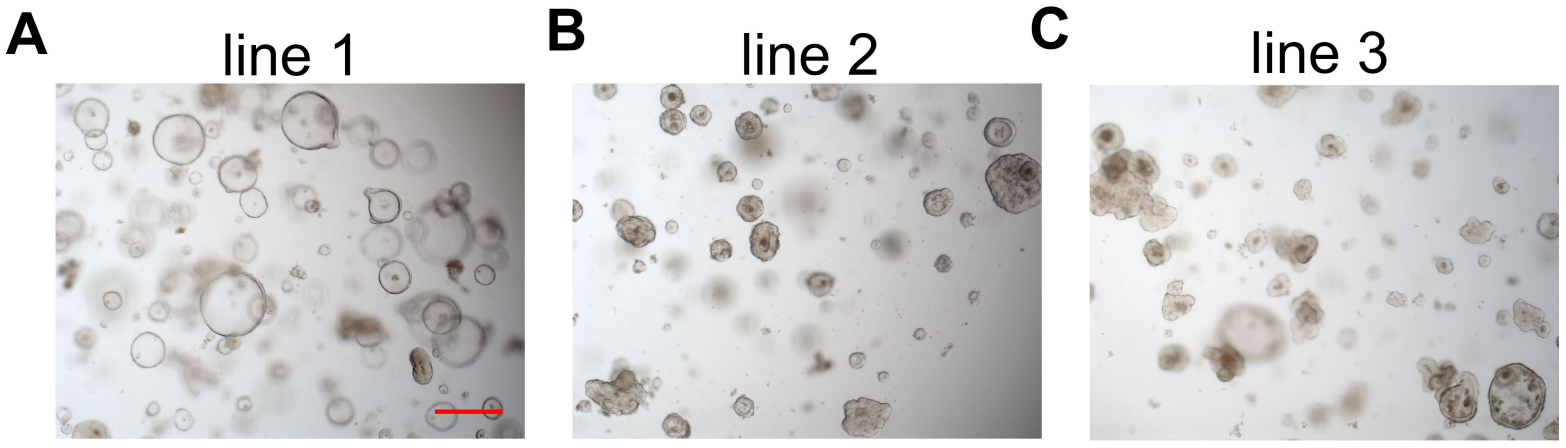
Bright-field images showing the phenotypes of three rectal cancer organoid lines. **(A)** Organoids (line 1) with a hollow architecture. **(B)** Organoids (line 2) with a more solid architecture. **(C)** Organoids (line3) with a mix architecture with hollow and solid organoids. Scale bar, 100 μm.

**Figure 2:**
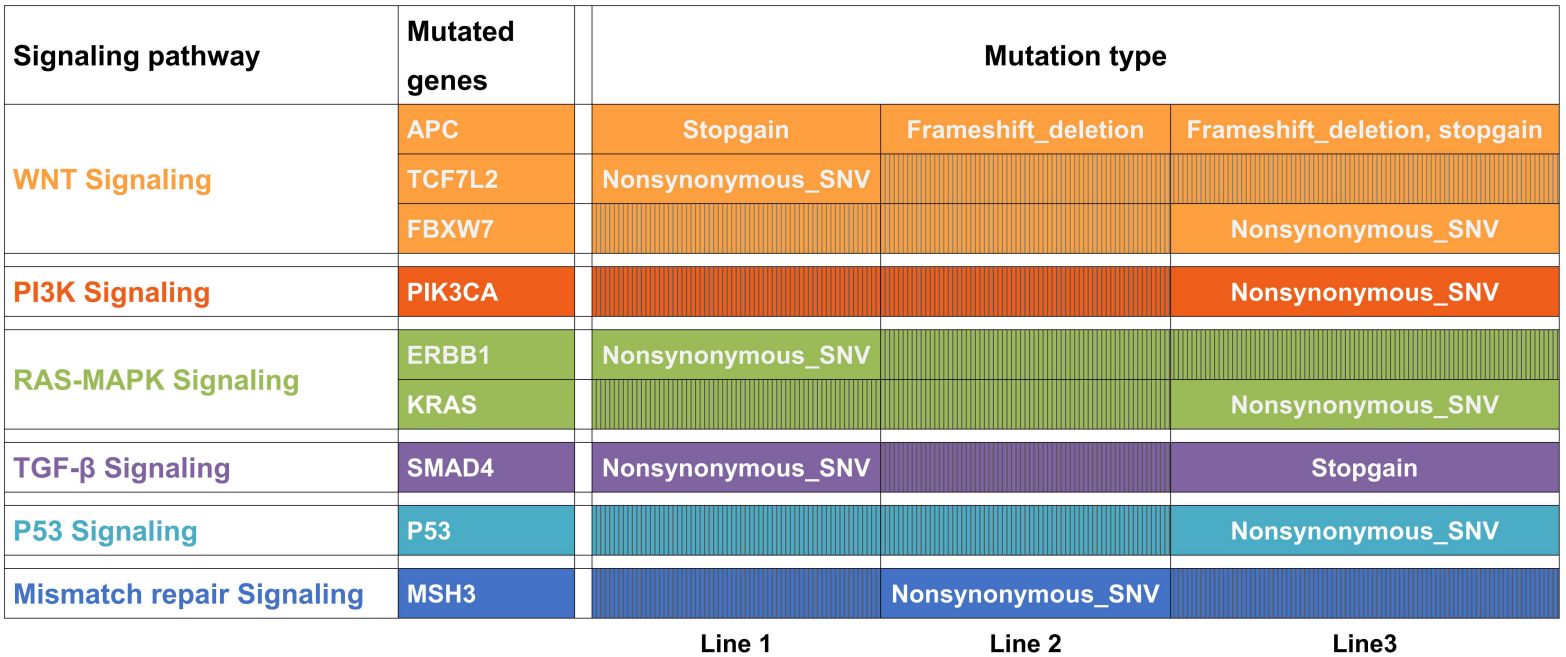
Most frequently mutated genes among rectal cancer tumors were present in the three organoid lines. Some of these genes were mutated in line 1 (APC, TNN, SMAD4, TCF7L2, ERBB2 and MAP7), line 2 (APC, TNN and MSH3) and line-3 (APC, TNN, TP53, FBXW7, KRAS, SMAD4 and PIK3CA). Most of these genes are involved in the WNT, PI3K, RAS-MAPK, TGF-β, and P53 signaling pathways, which are altered in colorectal and rectal cancers. M means mutation.

In this study, we selected three lines of rectal cancer organoids derived from patients for further research. All three patients had high-lying rectal cancer. Patient 1 had a postoperative pathological stage of pT2N0M0, was assessed as low risk after surgery, and did not receive adjuvant treatment. Patients 2 and 3 both had a postoperative pathological stage of pT3N0M0, were also assessed as low risk after surgery, and did not receive adjuvant treatment. Surgical specimens were obtained from patients with newly diagnosed rectal cancer under informed consent. The pathological type of all three organoid lines was adenocarcinoma. Surgical specimens were minced with a surgical knife, and tumor cell aggregates were isolated through enzymatic digestion and mechanical disruption. The isolated tumor cells were seeded into Matrigel and cultured in organoid culture medium (see *Materials* and *Methods*). One organoid line (line 1, moderately differentiated, G2) displayed a hollow architecture (**Figure 1A**); line 2 (moderately to well differentiated, G1–G2) displayed a more solid architecture (**Figure 1B**); and line 3 (moderately to poorly differentiated, G2–G3) exhibited a mixed architecture with both hollow and solid organoids (**Figure 1C**). This illustrated the heterogeneity of the tumors. The heterogeneity of the organoids stems from the heterogeneity of tumors derived from different patients. From another perspective, the heterogeneity of tumor organoids may be driven by distinct genetic variations. Regarding the heterogeneity of patient-derived tumor organoids, we investigated this in our study published in Cell Stem Cell in 2020 (**Figure 2**) (19). It has been well confirmed that the histopathological features of organoids and primary tumor tissues are similar (14, 24, 29, 30).

### Genetic characterization of rectal cancer organoids

We performed whole-exome sequencing (WES) to characterize the mutational features of the three organoid lines. The most frequently mutated genes among rectal cancer tumors have been well profiled (31). We found that some of these genes were mutated in line 1 (APC, TNN, SMAD4, TCF7L2, ERBB2, and MAP7), line 2 (APC, TNN, and MSH3), and line 3 (APC, TNN, TP53, FBXW7, KRAS, SMAD4, and PIK3CA). Most of these genes are involved in the WNT, PI3K, RAS–MAPK, TGF-β, and P53 signaling pathways, which are frequently altered in colorectal and rectal cancers (**Figure 2**). Our results revealed that WNT signaling was the most frequently mutated pathway in rectal cancer, confirming the importance of the WNT pathway (**Figure 2**). Similar to previous research (31), other alterations in the WNT signaling pathway often occurred in organoids harboring APC mutations, suggesting that multiple mutations in WNT pathway components might confer a selective advantage (**Figure 2**). Consistent with previous reports, our results suggest that rectal cancer organoid lines recapitulate the mutational features of rectal cancer and therefore represent a reliable model for investigating treatment responses.

Considering the composition of the culture medium, which lacked WNT3A, and the genetic characterization described above, we can conclude that the successfully established organoid lines were rectal cancer organoids rather than organoids derived from normal rectal epithelium.

### Dose response of rectal cancer organoids to irradiation tested by cell viability

**Figure 3:**
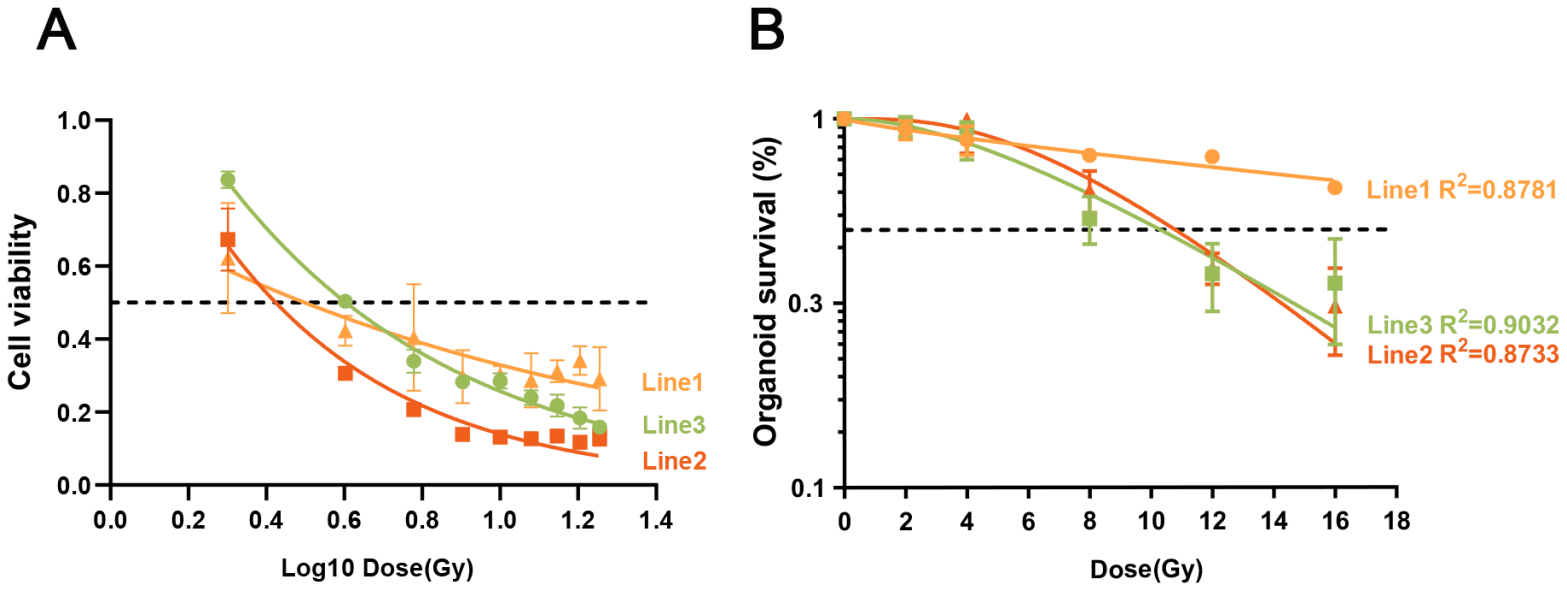
Dose response of rectal cancer organoids to irradiation tested by cell viability and image-based analysis. **(A)** Dose–response curves based on cell viability showed different sensitivities of the three organoid lines to irradiation. Organoid line 1 was less sensitive, line 2 was the most sensitive, and line 3 was intermediately sensitive. Cell viability data are shown as means ± SD from triplicate samples. The dotted line indicates 50% organoid survival. **(B)** Dose response curves tested by image-based analysis showed different sensitivity of the three organoid lines to irradiation. Organoid line 1 was less sensitive, line 2 and 3 were more sensitive. Surviving organoids data are shown as means ± S.E from three independent experiments (n=12). Dotted line means the rate of organoids survival is 50%.

Cancer organoids have recently been used for drug screening in translational medicine research (24, 30, 32, 33). In these studies, the dose response of cancer organoids to anticancer drugs was mainly evaluated using ATP-based cell viability assays. Here, we used the CCK-8 assay to test the dose response of the three rectal cancer organoid lines to irradiation. Cell viability was measured on day 9 after irradiation (0, 2, 4, 8, 12, and 16 Gy). The irradiation dose-response curve fitting utilized the log[inhibitor] vs. normalized response-variable slope model with the equation Y = 100/(1 + 10^((LogIC50-X)*HillSlope)) for results obtained from the CCK8 assay. The dose-response curves showed different sensitivities of the three organoid lines to irradiation (**Figure 3A**). Organoid line 1 was the least sensitive (IC50 = 1.495e-006), line 2 was the most sensitive (IC50 = 0.01094) and line 3 showed intermediate sensitivity (IC50 = 0.002921; **Figure 3A**). Notably, cell viability was significantly decreased after 2 and 4 Gy irradiation, from 100% to <84% and <51%, respectively, among all of the three lines (**Figure 3A**).

Panel B: Dose–response curves based on image analysis showed different sensitivities of the three organoid lines to irradiation. Organoid line 1 was less sensitive, whereas lines 2 and 3 were more sensitive. Surviving organoid data are shown as means ± SE from three independent experiments (n = 12). The dotted line indicates 50% organoid survival.

### Image based analysis of dose response of rectal cancer organoids to irradiation

For proliferating cells, survival or death was defined as the loss of the capacity for sustained proliferation—that is, loss of reproductive integrity (20). This is certainly the endpoint typically evaluated in proliferating cells cultured *ex vivo*. The *in vitro* dose–survival curve obtained from the colony formation assay serves as the basic model in cancer radiobiology (20). As described above, organoids may represent a more appropriate model for radiobiological research in cancer. At present, organoid cell death is mainly evaluated using cell viability assays, which do not adequately measure the reproductive integrity of cancer cells.

**Figure 4:**
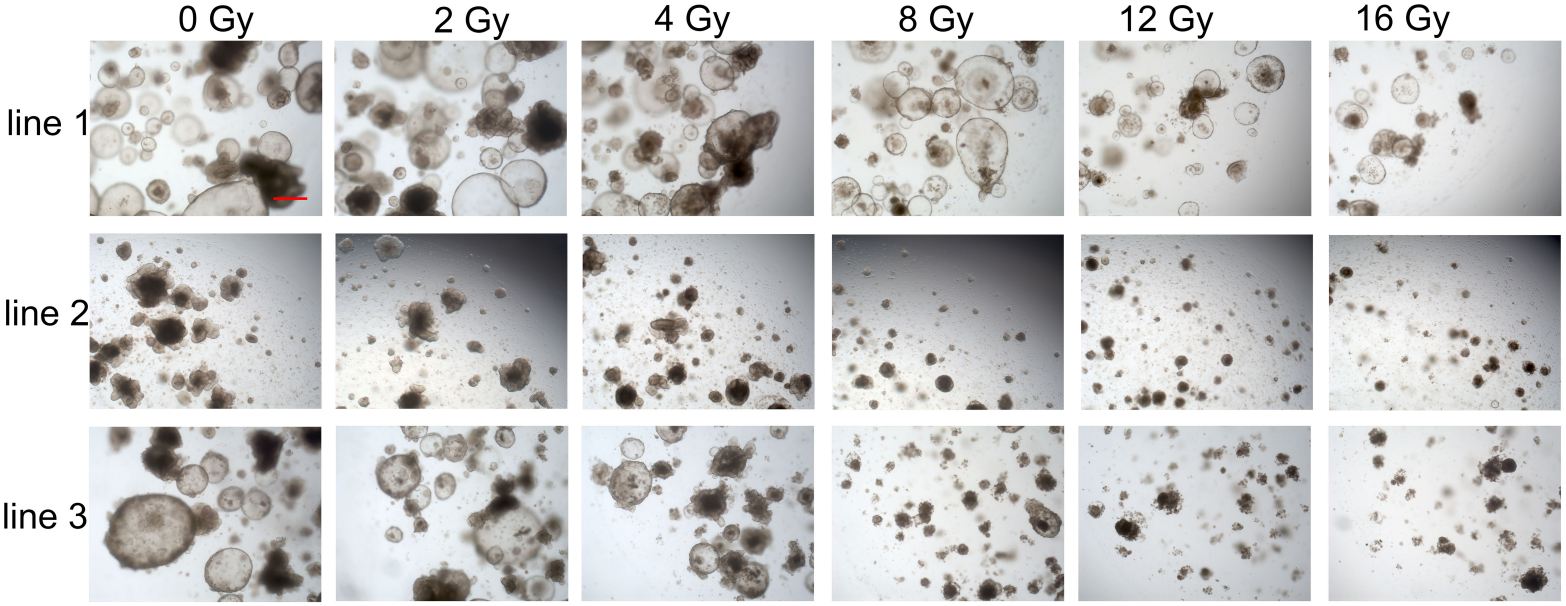
Representative bright-field images of organoids at day 15 after irradiation (0, 2, 4, 8, 12, and 16 Gy). Scale bar, 100 μm.

Here, we speculated that image-based analysis would be superior to cell viability testing for assessing organoid cell death after irradiation. To validate the *in vitro* response of rectal cancer organoids to irradiation, we analyzed organoid dose–response data at day 15 (0, 2, 4, 8, 12, and 16 Gy), evaluating survival rates on the basis of light microscopy imaging as described in *Materials* and *Methods*. Bright-field images of organoids after 0–16 Gy irradiation at day 15 are shown in **Figure 4**. Viable organoids maintained complete structures, whereas nonviable organoids exhibited disrupted structures (**Figure 4**).

The irradiation dose–response curve fitting utilized the single-hit multi-target model with the equation Y = 1-(1-exp(-k*x))^N (27, 28) for the results obtained from image-based organoid survival analysis. The dose–response curves again demonstrated different sensitivities among the three organoid lines (**Figure 3B**). Organoid line 1 was less sensitive, whereas lines 2 and 3 were more sensitive (**Figure 3B**). The corresponding radiosensitivity parameters are listed in **Table 1**.

**Table 1:** Radiosensitivity parameters of the three organoid lines.

Organoids	D0	Dq	N	SF2
Line 1	57.18	-12.40	0.81	93.37%
Line 2	6.18	7.93	3.61	99.04%
Line 3	8.18	5.97	2.08	95.81%

D_0_;, Dq, N, and SF_2_ represent the mean lethal dose, quasi-threshold dose, extrapolation number, and survival fraction at 2 Gy, respectively.

### Cancer organoids as a novel model for the evaluation of dose response to irradiation based on imaging analysis

As described above, for proliferating cells, loss of the capacity for sustained proliferation—or loss of reproductive integrity—is the key criterion for defining cell survival or death in radiobiology. Cell viability, therefore, is not suitable for analyzing cell death after irradiation.

Microscopic inspection (**Figure 4**) clearly showed that more than 50% of rectal cancer organoids in all three lines remained viable at day 15 after 4 Gy irradiation. Organoid lines 2 and 3 exhibited similar radiosensitivity and were both more sensitive than line 1. This observation was consistent with the survival curves obtained from image-based analysis (**Figure 3B**), where line 1 remained on top and lines 2 and 3 clustered closely below. In contrast, the cell viability assay (**Figure 3A**) failed to capture these differences: the survival curve of line 1 did not clearly demonstrate greater radioresistance compared with lines 2 and 3, nor did it reflect the comparable radiosensitivity of lines 2 and 3, resulting in a pattern inconsistent with the morphological findings.

Importantly, the image-derived survival curves also resembled the classical radiobiological profile, with an initial linear slope, a shoulder, and a subsequent straightening phase (**Figure 3B**) (20). Therefore, we concluded that image-based analysis is more reliable than cell viability assays for assessing organoid cell death after irradiation.

Based on our results, we recommend that image-based analysis methods be used when cancer organoids are employed in radiobiological research.

## Discussion

Cancer cell lines and patient-derived tumor xenografts (PDTXs) are the standard and most commonly used preclinical human cancer models (2). Cancer cell lines derived from patients have contributed greatly to cancer research, but they have many limitations, including a low initiation success rate, poor retention of phenotypic features *in vitro*, instability of genetic characteristics, and lack of tumor–stroma interactions (7). PDTXs are established by transplanting freshly derived human cancer tissues into immunodeficient mice and perform well in mimicking the genetic and biological characteristics of human tumors. However, establishing PDTXs is time-consuming and expensive, and the engraftment success rate is also very low (34).

As described above, organoids—especially cancer organoids—are emerging as a promising and novel tool in basic and translational medicine (8, 10). Oncologists have mainly focused on the potential value of organoids in basic cancer research and chemotherapy drug screening (7, 9). However, most radiation oncologists are still unaware of the value of organoids in radiation oncology research. The present study demonstrates that patient-derived cancer organoids have great potential in radiation oncology and that image-based analysis of organoids is superior to cell viability assays for assessing cell death after irradiation.

In this study, we emphasized the importance of image-based analysis for evaluating cell death in cancer organoids. Our results showed that cell viability did not accurately represent organoid survival. In traditional radiobiology, cells are considered to have survived when they grow into relatively large colonies that differ little from unirradiated controls, based on image analysis, although colony size may vary (20). Similarly, organoids are regarded as viable when their structures remain intact after irradiation, and image-based analysis remains the best evaluation method. To date, nearly all studies have used cell viability assays to assess cell death in cancer organoids for drug screening (10, 24, 29, 30). Therefore, it is worth emphasizing that cell viability assays are not appropriate for evaluating cancer organoids in radiation oncology research.

In our study, bright-field imaging was used for analysis, relying on our extensive experience in organoid research. Under certain circumstances, however, it can be difficult to determine whether organoids are viable based solely on their structures. We recommend that additional staining methods be used for further identification. For example, cell death in organoids can be assessed by live/dead staining (Calcein-AM/PI staining) followed by fluorescence confocal microscopy (26, 35). More importantly, live/dead staining facilitates the use of data analysis software (e.g., Image-Pro Plus) for automated data processing, which is especially useful when handling large datasets.

Our study had several limitations. First, we compared the efficacy of cell viability and image-based analysis for evaluating organoid cell death using only three representative lines. In addition, the dose response of organoids to chemotherapeutic drugs was not assessed. As is well known, chemoradiation is a common treatment for rectal and other cancers. Moreover, we did not validate whether organoids recapitulated the pathological features of tumor tissues as comprehensively as shown in other studies.

Patient-derived cancer organoids represent a major advancement over cancer cell lines, cancer spheroids, and PDTXs and provide a new, powerful, and promising system in cancer radiobiology. Beyond their biological reproducibility, the limitless lifespan and relatively low cost of cancer organoids are additional advantages. It is predictable that patient-derived cancer organoids will become a popular and effective tool for radiation oncologists in basic and translational research, particularly in precision radiation oncology. When using cancer organoids in radiation oncology research, it must be borne in mind that image-based analysis is superior to cell viability assays for evaluating organoid cell death.

## Conclusions

Our data from rectal cancer organoid lines revealed that cancer organoids recapitulate the mutational features of rectal cancer and can be applied to the assessment of dose response to irradiation. We also validated that image-based analysis is superior to cell viability assays for evaluating organoid cell death after irradiation. This research lays a methodological foundation for the application of cancer organoids in the field of cancer radiobiology.

## Data Availability

The data presented in the study are deposited in the Mendeley Data repository, 10.17632/sm83ff497n.1.
